# Decreased levels of physical activity in adolescents with down syndrome are related with low bone mineral density: a cross-sectional study

**DOI:** 10.1186/1472-6823-13-22

**Published:** 2013-07-04

**Authors:** Ángel Matute-Llorente, Alejandro González-Agüero, Alba Gómez-Cabello, Germán Vicente-Rodríguez, José Antonio Casajús

**Affiliations:** 1 GENUD (Growth, Exercise, Nutrition and Development) Research Group, University of Zaragoza, Ed. Cervantes Corona de Aragón St 42, 2nd floor, Zaragoza 50009, Spain; 2 Faculty of Health and Sport Science, Department of Physiatry and Nursing, University of Zaragoza, Ronda Misericordia 5, Huesca 22001, Spain; 3 Department of Sport and Exercise Science, Aberystwyth University, Ceredigion, United Kingdom; 4 Hospital Virgen del Valle, Complejo Hospitalario de Toledo, Toledo, Spain

**Keywords:** Osteoporosis, Osteopenia, Accelerometer, Dual energy x-ray, Actitrainer, Body composition

## Abstract

**Background:**

Down syndrome (DS) has been described as one of the main contributors for low bone mineral density (BMD). Physical activity (PA) is a key factor in skeletal health and thus, PA levels might be associated to the risk of developing osteoporosis. Therefore, the aims were (1) to describe PA patterns in adolescents with DS compared to their counterparts and (2) to determine the relationships between PA and the risk of having low bone mass in adolescents with DS.

**Methods:**

Nineteen adolescents (10 girls) with DS and 14 without disabilities (7 girls) participated in the study. Minutes in different PA intensities were objectively assessed with accelerometers (ActiTrainer). Moreover adolescents with DS were classified into PA tertiles taking into account the amount of total minutes of PA at any intensity, resulting in those performing low, medium or high of PA (lowPA, medPA and highPA). BMD was measured at the whole body, hip and lumbar spine with dual-energy X-ray absorptiometry and the BMD Z-score was calculated for each region taking into account age- and sex-matched reference data. Student’s unpaired t-tests and analysis of covariance were used to compare variables between different conditions (DS vs. control) and PA levels (low, medium and high).

**Results:**

None of the adolescents with DS achieved the minimum of 60 min of daily moderate to vigorous PA (VPA) intensity recommended by PA guidelines; adolescents with DS group spent less time in sedentary and in VPA and more time in light PA than those without DS (p < 0.05). Adolescents with DS showed lower BMD Z-score values than those without (p < 0.05). Those adolescents with DS allocated in the lowPA tertile showed significant lower BMD Z-score at the hip and a general tendency towards lower BMD Z-score was found at whole body and lumbar spine compared to those in highPA tertile and (p < 0.05).

**Conclusions:**

Adolescents with DS in the highPA tertile showed lower risk of developing future osteoporosis by having higher BMD Z-score at the hip. This data provides an idea regarding the importance of accumulated minutes of PA, and not only moderate or vigorous in the bone health in adolescents with DS.

## Background

Persons with Down syndrome (DS) have shown diminished levels of bone mass compared with those without DS [1-4]; therefore, DS population might be considered as a population at higher risk of suffering bone fractures and osteoporosis. The latter is a systemic skeletal diseases characterized by low bone mass and micro-architectural deterioration, resulting in an increased susceptibility to fracture [5]. Osteoporosis-related fractures constitute a major public health concern in the nowadays society [6]. Among the factors involved in fracture risk Lips et al. [7] highlighted bone mineral density (BMD), bone geometry and bone strength. Furthermore, as life expectancy in DS population has been increased over the last 70 years and this trend is expected to continue [8] the incidence of osteopenia and osteoporosis is likely to be augmented in this population within the coming years. In addition, and reinforcing this hypothesis, DS has been demonstrated as one of the main contributors for low BMD in persons with intellectual disability [9,10]. It is widely known that a increased bone mass acquisition during childhood and adolescence is determinant for a good skeletal health in adulthood [11]. Although genetic factors highly determine bone mass acquisition [12], environmental and lifestyle factors, such as physical activity (PA) have an important role in bone mass acquisition due to their osteogenic effects [13]. PA is a key factor in disease prevention and provides several benefits in overall health [14] and specifically in skeletal health [15]. Exercise has important osteogenic effect, mainly when high-impact and weight-bearing PA occur [16] in concordance with the Wolff’s law [17] which postulates that bones adapt to mechanical loads. At the same time, the mechanostat theory [18,19] suggests that both systematic exercise and PA could drive to a direct osteogenic effect on bone mass and an indirect osteogenic effect by increasing muscle size and strength and hence the tensions generated on bones [16]. Persons with DS experience several barriers to participate in daily PA like transportation restrictions, low motivation and lack of integrated program options [20]. Consequently, low levels of PA [21] and physical fitness [22] have been described in this population. In addition, most of the literature regarding PA levels in children with intellectual disabilities was based on self or proxy-reported questioners, or pedometers [23] with the consequent methodological weaknesses [24]. Even with that, there was some evidence indicating that children with intellectual disabilities are less active than those without disabilities [23]. Accelerometry is an accurate, non-invasive, and relatively low-cost method that can be used with minimal interference in free-living conditions [25]; and this has been demonstrated as more reliable than pedometers [26]. Some studies using accelerometers have been performed in order to evaluate PA levels in children with DS [21,27-29]. A general tendency towards decreased levels of PA with increasing age has been shown in different studies, and also a non-achievement of the currently recommendations of PA was found in this population. As observed, information concerning bone mass in young populations with DS is scarce [30] and should therefore be given greater attention; on the other hand PA levels might have a relationship with the risk of developing osteopenia or osteoporosis.

Therefore the aims of this study were (1) to describe PA patterns in adolescents with DS compared to their counterparts and (2) to determine relationships between PA and risk of having low bone mass in adolescents with DS.

## Methods

**Figure 1:**
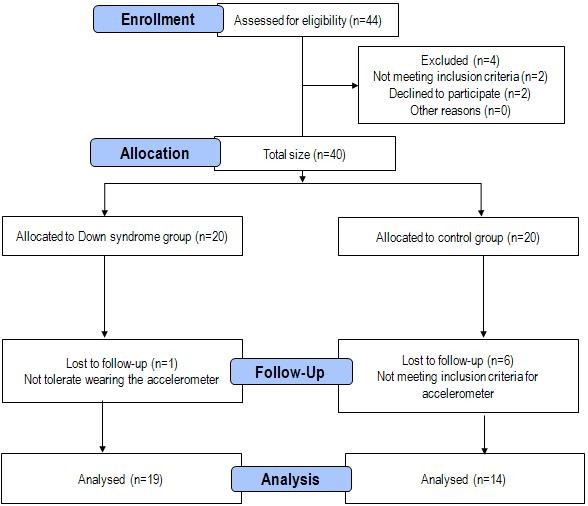
Consort flow diagram.

Institutions were contacted prior to the study to organize reunions in order to connect with parents and offer to participate in the study. Once the informed consents were obtained, 40 adolescents were selected taking into account their age, sex and the lack of contraindications (Figure 1). The contraindications for taking a DXA scan were: being pregnant or having had any procedure using substances such as Iodine, Barium, and Nuclear medicine isotope.

The present study was conducted on two groups: the first group was comprised by 20 adolescents with DS (10 girls, 10 boys; aged 14.7 ± 2.2 years) living at home, who were recruited from different special schools and institutions within the region of Aragon in Spain. Adolescents with DS were examined by an experienced cardiologist and all of them were healthy and given permission to participate in the study.

The second group was formed by 20 adolescents without DS (10 girls, 10 boys; aged 13.2 ± 2.8 years) and was also recruited from regular schools in the same region. All the adolescents without DS were healthy and without known illness, and all subjects had been medication-free for at least 3 months before the test. A full clinical history, including illnesses or surgical interventions and stays in a hospital, was collected for each individual. Both parents and children were informed about the aims and procedures of the study, as well as the possible risks and benefits, and then, a letter of written informed consent was obtained from all the included subjects and their parents or guardians. The study was performed in accordance with the Helsinki Declaration 1961 (revised in Edinburgh, 2000) and was approved by the Research Ethics Committee of the Government of Aragon (CEICA, Spain).

### Anthropometric measures

All subjects were measured with a stadiometer without shoes and the minimum clothes to the nearest 0.1 cm (SECA 225, SECA, Hamburg, Germany), and weighted to the nearest 0.1 kg (SECA 861, SECA, Hamburg, Germany) following the procedures by International Society for the Advancement in Kinanthropometry (ISAK) [31]. Body mass index (BMI) was calculated as weight (in kilograms) divided by height (square meters). Height, weight and BMI Z-scores were calculated using a Microsoft Excel add-in to access growth references based on the LMS method [32] using a reference European population [33].

### Pubertal status assessment

Pubertal development was determined by a sport medicine physician with direct observation according to the five stages proposed by Tanner and Whitehouse [34].

### Assessment of PA

Free-living PA was objectively assessed using the ActiTrainer uniaxial accelerometer (Actigraph, LLC, Pensacola, FL, USA). Actitrainer uniaxial accelerometer has been validated for assessing PA and energy expenditure [35]. ActiTrainer is a small and light device (8.6 × 3.3 × 1.5 cm, 51 g), which uses a sensor with a 0.25 to 2.5 g range capable of detecting movements in the vertical plane and a sampling frequency of 30 Hz. For this study, a 15s epoch was selected on the basis of literature recommendations [36]. The accelerometer was worn for seven consecutive days during school-term time and was mounted on the right hip of each adolescent by means of an elastic waistband. Instructions were given to the participants and caregivers both verbally and in writing on how to wear the accelerometer during all waking hours except while bathing, showering, swimming, and playing contact sports. A time sheet was used to register when the accelerometer was placed on or removed each day. In DS group, it was completed by parents or caregivers whilst in control group it was completed by themselves. A 7-day data collection period has been found to provide sufficient time to obtain a reliable estimation of normal activity in children and adolescents aged 6–17 years, as it allows for differences in PA levels across the day and between weekdays and weekends [37]. Afterwards, the previous published cut-off points proposed by Evenson et al. [38] and recommended by Trost et al. [39] were used to estimate time spent in sedentary, light-, moderate-, and VPA intensity in children and adolescents.

### Data reduction

For inclusion in this study, the accelerometer had to be worn for a minimum of 10 hours per day, for at least 4 days out of the 7-day monitoring period, including one weekend day, as recommended in a previous study [21]. Data analyses involved the data processed in the R [40] program. The methodological process of this program has been described in a previous research [35]. Based on a 15 s epoch, the data were then reduced and assigned to one of the following categories: sedentary activity (counts ≤ 25), light PA (574 > counts > 25), moderate PA (1003 > counts of ≥ 574), or VPA(counts ≥ 1003). Subjects were considered compliant to the PA guidelines if their moderate to VPA, averaged over the valid days of monitoring, was ≥ 60 min a day [41]. In our study, total minutes of daily PA is equivalent to valid time being the average daily time spent wearing the accelerometer once the data reduction was done according to Ojiambo et al. [42]. A 20-min period of zero counts is produced when the accelerometer device records 20 minutes of inactivity, that is, 20 minutes of 0 counts. During data reduction 20-min period of zero counts were automatically deleted with the R program.

### Bone mass

Bone mass of the participants was measured with dual-energy X-ray absorptiometry (DXA) using a pediatric version of the software QDR-Explorer (Hologic Corp. Software version 12.4, Waltham, MA). DXA equipment was calibrated daily with a lumbar spine phantom and step densities phantom following the Hologic guidelines as recommended the manufacturer. Subjects were scanned in supine position, and the scans were performed in high resolution. BMD (in grams/cm^2^) was obtained from the whole body scan, lumbar spine (L_1_-L_4_) and proximal region of the femur (hip). BMD Z-score values were calculated for purposes of comparison. The BMD Z-score is a measurement which compares the actual BMD of a person with age- and gender-matched reference values obtaining a score of SD from the reference M. This is a more appropriate measurement for bone health in children and adolescents than t-score is [43].

### Statistical analysis

All data analyses were performed using SPSS 15.0 software for Windows (SPSS Inc. Chicago, IL), and significance was set at *p*≤ 0.05. Mean (M) and Standard Deviation (SD) or Standard Error (SE) are given as descriptive statistics; otherwise they are stated. All variables included in this study showed a normal distribution, assessed by Kolmogorov-Smirnov test. Differences between DS and control groups for age, and height, weight, BMI, BMD Z-scores were established using the Student’s unpaired *t*-tests. Analyses of covariance (ANCOVA) were performed to evaluate differences between DS and control groups in daily minutes in different PA intensities, with age and valid time as covariates.

Tertiles of total PA, based on the total minutes of daily PA: lowPA (min ≤ 713), medPA (795 ≥min > 713), and highPA (min > 795), were also calculated within the group of adolescents with DS. ANCOVA was also performed in order to evaluate differences in BMD Z-score values between different tertiles of PA (with further Bonferroni post-hoc test) entering Tanner stage, height and whole body lean mass as covariates.

## Results

### Participants

Final reduction of accelerometry data resulted in 7 participants lost for different reasons (see Figure 1).

### Physical characteristics

Age and physical characteristics are summarized in Table 1. No differences between groups were observed for age, weight, height and BMI. On average, participants with DS were older (M = 14.78, SE = 0.51), lighter (M = 46.76, SE = 2.65) and smaller (M = 146.30, SE = 2.84) than those without DS (M = 13.28, SE = 0.75), (M = 50.23, SE = 4.20) and (M = 155.57, SE = 3.99). These differences were not significant *t*(31) = −1.69, 0.73, 1.94, (all p > 0.05) (Table 1); these values did represent a medium-sized and small-sized effect r = .32, .13 and .33 respectively. When weight, height and BMI Z-scores were taken into account, adolescents with DS showed significantly differences in Height (M = −2.30, SE = 0.25), and Weight (M = −0.83, SE = 0.27) Z-scores compared with those adolescents without DS (M = 0.00, and 0.35, SE = 0.25, and 0.34 respectively) *t*(31) 6.4 and 2.7, (all p < 0.05) (Table 1); and they represent a high and medium-sized effects r = .75, .44. However, there was no difference in BMI Z-score values between DS group (M = 0.62, SE = 0.20) and Control group (M = 0.46, SE = 0.33) *t*(31) -0.41, (p > 0.05) (Table 1).

**Table 1:** **Descriptive statistics for participants** (**M** ± **SD**)

	**Down syndrome**	**Control**
	**(n = 19)**	**(n = 14)**
Age (yr)	14.7 ± 2.2	13.2 ± 2.8
Weight (kg)	46.7 ± 11.5	50.2 ± 15.7
Height (cm)	146.3 ± 12.3	155.5 ± 14.9
Body mass index	21.4 ± 3.2	20.3 ± 3.7
Weight Z-score	−0.8 ± 1.2*	0.4 ± 1.3
Height Z-score	−2.3 ± 1.1*	0.0 ± 0.9
BMI Z-score	0.6 ± 0.9	0.5 ± 1.3
Lumbar spine BMD Z-score	−0.8 ± 0.9*	0.1 ± 1.1
Hip BMD Z-score	−1.1 ± 0.9*	0.1 ± 1.2
Whole body BMD Z-score	−1.7 ± 1.1*	0.5 ± 1.6

* *p* ≤ 0.05 between groups. *BMD* bone mineral density.

### Physical activity

**Figure 2:**
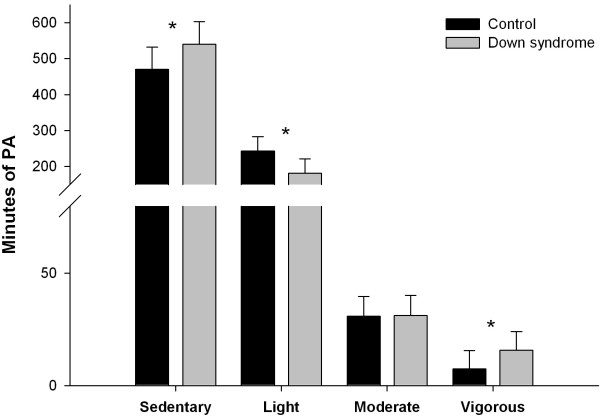
**Daily time spent in different PA intensities adjusting by age and valid time.** * *p* ≤ 0.05 between groups.

Total minutes in each PA intensity adjusting by age and valid time are presented in Figure 2. There was a significant effect of the age and the valid time on sedentary PA, F (1, 29) = 21.80, (p < 0.05) and F (1, 29) = 7.31, p < 0.05. Bonferroni *post hoc* test revealed that the sedentary PA was significantly lower in DS group (M = 470.7, SD = 61.3) than in control group (M = 540.2, SD = 62.3); (p < 0.05). In light PA, age F (1, 29) = 43.45, (p < 0.05) and valid time F (1, 29) = 7.38, (p < 0.05) had a significant effect on it. However, in this case DS group (M = 243.5, SD = 39.5) spent more time light PA than control group (M = 181.1, SD = 39.8); (p < 0.05). In moderate PA, only age F (1, 29) = 29.8, (p < 0.05) had a significant effect on it; but valid time F (1, 29) = 0.1, (p > 0.05) do not and Bonferroni *post hoc* test did not reveal any different. However, adolescents with DS (M = 7.4, SD = 8.2) engaged less time in VPA than those without (M = 15.8, SD = 8.4) but neither age F (1, 29) = 0.02 nor valid time F (1, 29) = 0.65 had a significant effect on VPA (all p > 0.05) (See Figure 2).

None of the adolescents with DS achieved the minimum of 60 min of daily moderate to VPA recommended by guidelines; while only 3 of the control adolescents achieved that amount, which represents 21.4% of the sample (data not shown).

### Bone mass

Adolescents with DS showed lower BMD Z-score values at the whole body (M = −1.75, SE = 0.24), lumbar spine (M = −0.83, SE = 0.20), and hip (M = −1.08, SE = 0.20) than the control group (M = 0.53, SE= 0.44), (M = 0.09, SE = 0.30) and (M = 0.08, SE = 0.33). All differences were significant *t*(31) = 2.64, *t*(30) = 3.18 and *t*(31) = 4.48, (all p < 0.05) (Table 1); and they represent a medium-sized and large-sized effects r = .43, .50 and .63.

**Figure 3:**
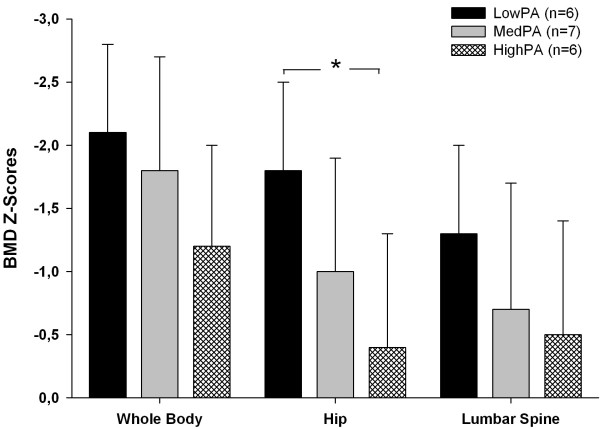
**Bone data for PA tertiles in the DS group.** Analysis adjusted for height, tanner stage and whole body lean mass. *p ≤ 0.05 between lowPA and highPA tertiles.

In DS group, there was a non-significant effect of height F (1, 13) = 0.12, and whole body lean mass F (1, 13) = 1.25 on BMD Z-score at hip region but tanner stage was F (1, 13) = 4.56, (p < 0.05). Once the sample of adolescents with DS was classified within PA tertiles, Bonferroni *post hoc* test revealed that those allocated in the lowPA tertile (M = −1.79, SD = 1.3) showed lower BMD Z-score at the hip region than those in highPA tertile (M = −0.45, SD = 1.5); (*p* ≤ 0.05) (Figure 3). Despite no significant differences between tertiles, a tendency towards higher BMD Z-score in medPA and highPA compared to lowPA was observed in lumbar spine and whole body BMD Z-score values (Figure 3).

## Discussion

The main finding of the present study is that adolescents with DS who engage daily in more minutes of total PA present higher BMD Z-score values, mainly at the hip region. Although some previous studies described PA patterns in children and adolescents with DS [21,27-29], to our knowledge, this is the first study including objectively assessed PA and bone measurements in adolescents with and without DS. Previous studies have found lower bone mass among individuals with DS compared with others without using different equipments such as DXA [2,3,44,45] or peripheral quantitative computed tomography [4]. Our study indicates, by means of lower BMD Z-score in all studied regions, higher risk of having low bone mass of the adolescents with DS, and, as the life expectancy of persons with DS has increased [8], this is an important issue to be taken into account. Literature indicates a consistent long-term protective effect from PA during adolescence on bone health [46] and also that sedentary behavior during childhood is associated with poor adult health outcomes [47]. In concordance, the results of our study showed that those adolescents with DS performing more minutes of daily PA had higher BMD Z-score values, especially at hip, than those engaging less minutes of daily PA. This relationship makes us believe that total minutes of PA could be a protective factor against poor bone-health. In addition to this, a recent published study has showed that the pelvis may be the first site to show significant differences in bone mineral content and BMD between preadolescent boys with and without DS [48]. This study has reinforced the importance of this body region in relation to bone health y DS population. Therefore, PA should be promoted in adolescents with DS, not necessarily at a high intensity, in order to decrease their risk of present and future low bone mass. Adolescents with DS in the current study engaged in less minutes of sedentary PA than those without; and in addition, the average values of the adolescents with DS in our study are lower than those observed by Esposito et al. [21]. At the same time, adolescents with DS engaged in more min of light PA than those without. However, adolescents with DS engaged in less minutes of high-intensity PA such as VPA, than those without DS. A paternal overprotection in adolescents with DS might be influencing and could partially explain these results [49]. Total minutes of VPA observed in DS adolescents in our study are in concordance with those showed by Phillips et al. [27] in their sample of adolescents with DS, but are far from those achieved by children with DS in other studies [28,29]. This fact might be also explained in part due to the general trend of decreasing PA with age in individuals with DS [21,27,29]. None of the adolescents with DS in the current study met PA recommendations of at least 60 min of daily MVPA, which is in agreement with some previous research [21,27], but in contrast with others [28,29]. Our results suggest that many children with DS may not perform enough PA to maintain an overall, and specifically bone good health. In fact, a recent study [13] reported that more than 78 minute of moderate to VPA per day are needed to increase bone mass in non-DS adolescents, which is farer from the actual MVPA of young DS population. This finding emphasizes the need of adolescents with DS to increase the amount of daily MVPA, as they are predisposed to diseases associated with inactivity such as osteoporosis, artery disease or obesity in adulthood [50]. The effect that the cut-off points choice has in the results obtained is something crucial [42]. Evidence suggests that the choice of epoch and cut-off points may interact and influence PA classification in an unexpected manner. Furthermore, Reilly et al. [51] re-analyzed data using different epochs and cut-off points for sedentary time and MVPA and found values ranging from 180 to 501 min of sedentary time per day and 28 to 266 min of MVPA per day for the same periods and individuals. In addition, Ojiambo et al. [42] found that the choice of different epochs had a significant effect on the time spent engaged in sedentary or MVPA. Therefore, attention might be put on this regard, and efforts should be made in order to evaluate which cut-off points better adapt to the characteristics of adolescents with DS, or whether it could be good to develop others specifically designed for this specific population. This study is not exempt of limitations which may affect the application of these findings. PA may have been underestimated as the use of a single, waist mounted, uni-axial accelerometer will not measure PA during upper-body and non-weight bearing activities (e.g., load carrying, swimming, cycling) and during activities such as bathing, showering, swimming, or playing contact sports. To complete the information regarding total daily physical activity, in future studies would be recommended to record the time in which participants were involved in swimming/ contact sports, slept, and showered/ bathed. In addition, the use of cut-off points established for children without disabilities may not be representative in individuals with DS as pointed out by Mendoca et al. [52]. Moreover, because the results are cross-sectional, a cause-and-effect relationship between PA and bone can only be suggested. The power to detect differences between groups may be affected by the small sample size.

## Conclusions

The current research provides evidence that adolescents with DS who perform more minutes of PA have lower risk of having low BMD at the hip and might be at lower risk of suffering osteoporotic fractures in the future by having a higher BMD Z-score in this region. Adolescents with DS did not achieve PA recommendations, and they engaged less minutes of VPA than those without DS. To develop specific cut-off points and/or PA recommendations for individuals with DS is an important topic to be taken into account in further research. These data remark the importance of total minutes of PA, and not only, those minutes in moderate or VPA, accumulated during the day in the population of adolescents with DS. Therefore, efforts to increase the PA levels in this population might be counteracting their intrinsic diseases of low bone mass, and preserving their future bone health.

## Abbreviations

DS: Down syndrome; BMD: Bone mineral density; PA: Physical activity; VPA: Vigorous physical activity; BMI: Body mass index; DXA: Dual-energy X-ray absorptiometry; M: Mean; SD: Standard deviation; SE: Standard error; ANCOVA: Analyses of covariance.

## Competing interest

The authors declare that they have no competing interest.

## Authors’ contributions

AML collected the accelerometers data, performed DXA scans and drafted the manuscript. AGA collected the accelerometers data, performed DXA scans, made the statistical analysis and drafted the manuscript. AGC performed DXA scans and drafted the manuscript. GVR and JAC designed and coordinated the study, and drafted the manuscript. All authors read and approved the final manuscript.

## Authors’ information

AML has a MSc and a predoctoral grant.

AGC has a PhD and a post doc position at Hospital Virgen del Valle, Toledo.

AGA has a PhD and is a Lecturer at Aberystwyth University.

GVR has a PhD and is a Reader at the University of Zaragoza.

JAC is MD with a PhD and is a Professor at the University of Zaragoza.

## Pre-publication history

The pre-publication history for this paper can be accessed here:

http://www.biomedcentral.com/1472-6823/13/22/prepub
